# Accuracy of Self-Estimated BMI in the Context of Telephone Assessment Before Elective General Surgery

**DOI:** 10.7759/cureus.37264

**Published:** 2023-04-07

**Authors:** Ashim Chowdhury, Charlotte Burford, Ellen Ainger, Roland Fernandes

**Affiliations:** 1 General Surgery, East Kent Hospitals University NHS Foundation Trust, Ashford, GBR

**Keywords:** preoperative planning, body mass index, covid-19, bmi, telemedicine

## Abstract

Background

This study aimed to determine if self-estimated body mass index (BMI) from telephone consultation was accurate and useful for surgical planning prior to elective general surgery.

Methods

A prospective cohort study was performed under a single surgeon at a district general hospital in the United Kingdom. Estimated BMI was collected from consecutive patients attending a pre-operative telephone consultation. Actual BMI was measured on the day of surgery and compared. Patient age and gender were also collected.

Results

Data were collected from 124 participants (median age 59 years, 49.2% male). A total of 33 participants under-estimated, 53 over-estimated, and 38 accurately estimated their BMIs. The median change in BMI was 0.0 (IQR -0.1, 0.3, p = 0.003). The median change in males was 0.0 (-0.1, 0.2, p = 0.479) compared to 0.1 (0.0, 0.7, p = 0.002) in females. Those with an actual BMI > 29.9 had a significantly higher median change (0.2 {0.0, 1.1}) compared to those with BMI ≤ 29.9 (0.0 {-0.2, 0.1}; p <0.001). Only two patients could have required a change in surgeon on the day of the procedure and this was not statistically significant (p = 0.500).

Conclusions

Self-estimated BMI, collected via telephone consultation, is a suitable method for assessing patients for surgical planning ahead of elective general surgery procedures, particularly for males. However, it is important to be aware that those with higher BMIs, particularly females, may underestimate their BMIs.

## Introduction

The coronavirus disease 2019 (COVID-19) pandemic placed a number of challenges on healthcare practice. One such challenge was to develop methods for delivering high-quality patient care without exposing already vulnerable groups to high-risk individuals (healthcare professionals) and high-risk environments (hospitals and medical centers). Telemedicine provided a feasible solution to this challenge and was adopted across the spectrum of clinical specialties during the pandemic [1]. The successful implementation of remote telephone and video consultations is likely to remain within clinical practice moving forward, even as restrictions around social distancing are eased [1].

Within surgical specialties, telephone consultations have been used successfully for outpatient appointments, including for preoperative consultations [2,3]. In general surgery, it is important to know a patient’s body mass index (BMI) prior to operating in order to plan for hospital bed space (day case or inpatient stay), theatre time (higher BMI is related to longer operating time), theatre equipment (need for long-handled instruments, larger operating beds, and specialized tilting facilities), and grade of surgeon (BMI over 35 usually requires a consultant to perform the surgery) [4]. Patients who are seen preoperatively in an outpatient clinic setting can have their BMIs measured at that time. However, during the COVID-19 pandemic, patients were asked to self-estimate their BMIs during a preoperative telephone consultation at our unit.

Given the increased use of telemedicine is likely to remain in clinical practice, we set out to understand if self-reported BMI was an accurate reflection of actual BMI and whether this was an appropriate measure to use for preoperative planning in general surgery.

## Materials and methods

This was a prospective cohort study carried out in a busy general surgery department at a district general hospital in the South-East of England. Consecutive patients attending telephone consultations, with a single upper gastrointestinal (UGI) surgeon at our unit over a six-month period between April and October 2021, ahead of elective general surgery were asked to self-estimate their BMI. Telephone consultations were conducted seven days prior to the date of the operation. On the day of the procedure actual height and weight were measured and "actual" BMI was calculated. A change in BMI was calculated to assess the difference between the estimated and actual BMI. Demographic data on patient age and gender were collected but no patient identifiable information was recorded.

Ethical approval was not sought as this study constitutes a service evaluation to determine if self-reported BMI is an accurate method for surgical planning prior to elective general surgery. Data were collated and analyzed using SPSS version 27.0 (Armonk, NY: IBM Corp.). Normality testing was used to determine appropriate descriptive statistics, and estimated and actual BMI were compared using non-parametric paired testing. The McNemar test was used to determine if surgical planning would have changed once the actual BMI was known. A p-value of < 0.05 was considered significant.

## Results

**Figure 1:**
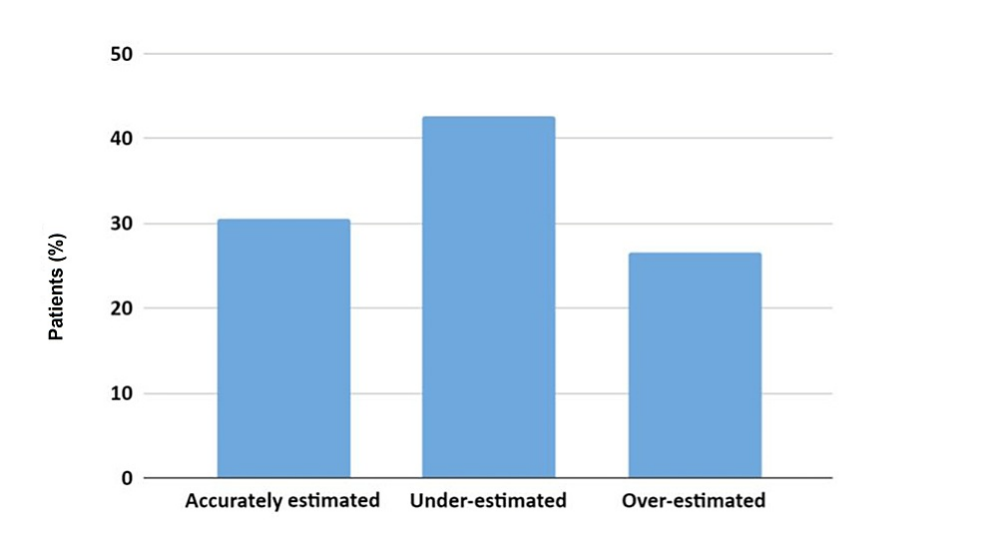
Accuracy of self-estimated BMI. The image shows the percentage of patients who (a) accurately estimated (b) under-estimated, and (c) over-estimated their BMIs.

There were 124 participants in the study, with a median age of 59 (IQR: 40.3, 71.0) years. A total of 49.2% of the cohort was male. There were 33 participants who under-estimated their BMI, 38 participants who accurately estimated their BMI, and 53 participants who over-estimated their BMI (Figure 1). The median change in BMI rating from estimated to actual for the whole cohort was 0.0 (IQR: -0.1, 0.3) and this was statistically significant (p = 0.003).

**Figure 2:**
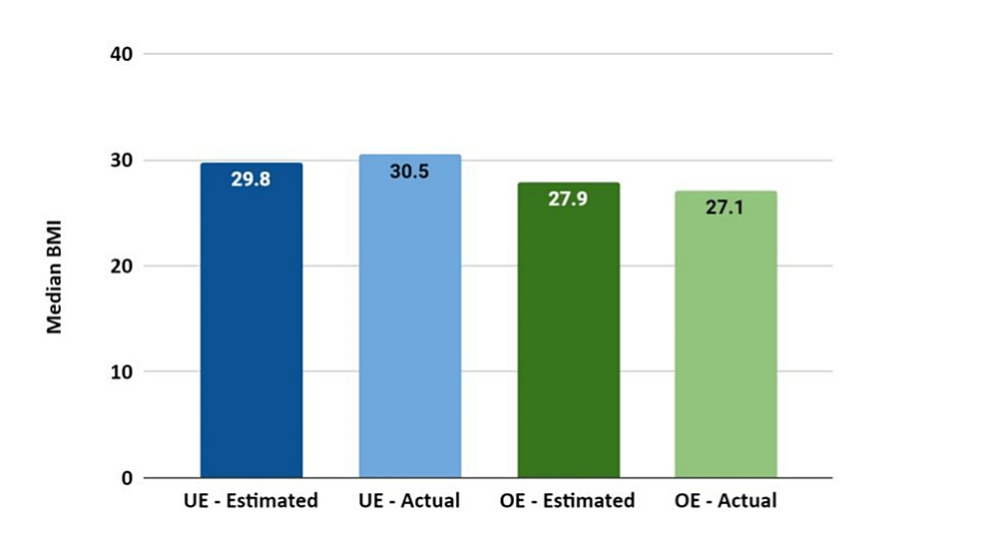
Median estimated and actual BMI of patients who under-estimated and over-estimated their BMIs.

The median estimated and actual BMIs for patients who under-estimated and over-estimated BMIs are shown in Figure 2. Of those who under-estimated their BMIs, the median difference in BMI was 0.3 (IQR: 0.2, 1.3; p < 0.001) while in those who overestimated their BMI, the median difference was -0.2 (IQR: -0.4, -0.2; p = 0.001). 

When comparing males and females, the males had a lower actual BMIs on average than females (26.1 {22.5, 30.3} vs 29.6 {26.1, 35.1}, p = 0.005) and a lower estimated BMI on average (26.8 {22.4, 30.3} vs 29.6 {26.4, 33.3}, p = 0.009). The median change in males was 0.0 (-0.1, 0.2, p = 0.479) compared to 0.1 (0.0, 0.7, p = 0.002) in females.

When comparing those with an obese actual BMI (>29.9) to those with a normal or overweight actual BMI (≤29.9) there was a significant difference in the median change (between estimated vs actual) of 0.2 (0.0, 1.1) compared to 0.0 (-0.2, 0.1, p < 0.001).

**Figure 3:**
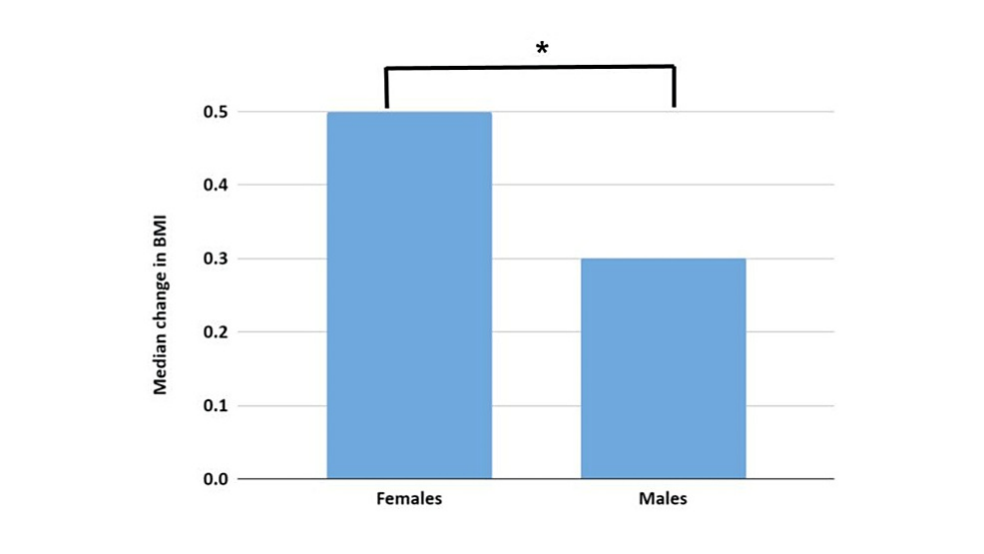
Gender differences in BMI estimates. The image shows the median change in BMI for males and females in those who under-estimated their BMIs. *Significant difference, p < 0.001.

Of those who under-estimated their BMIs, women significantly under-estimated their BMIs more (p = 0.037) than men with a median change between estimated and actual BMIs of 0.5 (IQR 0.25, 1.55) compared to 0.3 (IQR: 0.2, 0.4; p < 0.001) (Figure 3).

A cut-off of BMI >35 is often arbitrarily used as an indication of difficulty in certain procedures, such as laparoscopic cholecystectomy. As such these procedures are usually, but not exclusively, performed by a specialist consultant surgeon. In this cohort, there were 22 patients who would have needed a specialist consultant surgeon based on their estimated BMIs but 24 who needed one based on their actual BMIs. This difference was not statistically significant (McNemar, p = 0.500). The patients who could have needed a different surgeon on the day of the procedure were both female patients who estimated their BMIs to be between 30 and 35 but both had actual BMIs over 35.

## Discussion

This was a small prospective cohort study aimed at understanding whether self-estimated BMI can be used as an accurate measure in the context of telephone assessment prior to elective general surgery. Gall bladder surgery is most performed general surgical operation. Patients with a high body mass index (BMI) are at increased risk of having gallstones and are often considered at high risk of surgical complications due to their increased BMIs. The National Health Service (NHS) has rapidly adopted telemedicine solutions as an alternative to face-to-face consultations during the COVID-19 pandemic. The most common barrier in remote consultation was the inability to access patients.

Overall, the median change between the estimated and actual BMIs was zero. However, this is because participants both under- and over-estimated their BMIs. There was a significant difference between the estimated and actual BMIs (p = 0.003) despite the overall change being zero. This suggests that participants were not accurately estimating their BMIs. It has been previously shown that people are not accurate at estimating BMIs, with those with high BMIs typically over-estimating and those with lower BMIs under-estimating the true value [5].

In our cohort, the males undergoing elective general surgery procedures had a significantly lower actual BMI than females. The median change between estimated and actual BMIs was 0.0 for males and this was not significant. This suggests that males are accurate at estimating their BMIs. However, females showed a significant difference between estimated and actual BMIs with a median change of 0.1 suggesting females tend to under-estimate BMI.

Those who had an actual BMI above the cut-off for "obese" (>29.9) had a higher change between estimated and actual BMIs compared to those with a lower actual BMI (≤29.9). This suggests that those with higher BMIs are less accurate at estimating their BMIs and this makes operative planning somewhat more complex in terms of possible equipment required, manual handling, and operative times. Surgeons should be more cautious when planning for patients who give estimated BMIs over 30.

Of all participants who under-estimated their BMIs, not just those with a higher BMI, there was a greater under-estimate in females compared to males (0.5 vs 0.3) and this was statistically different. This suggests that women are under-estimating their BMIs more than men and so again a greater degree of caution may be needed for female patients.

Although several of our findings show statistical significance, it was felt by the authors that these statistical differences have a lower clinical significance. For example, the greatest average change we observed between estimated and actual BMI was for females who under-estimated their BMIs and this was only 0.5. Although operative time may increase in those who have higher than expected BMIs, it is unlikely a change of a couple of BMI points would make a difference to the estimated window of operating time required by the surgeon [4]. However, it is recommended that in patients with BMI > 35 undergoing a laparoscopic cholecystectomy consideration may be given to the procedure being performed by a dedicated UGI surgeon, given the potential for added complexity. It is therefore possible that an inaccurate BMI could lead to the need for a change in surgeon on the day of the procedure. In this cohort, there were only two cases in which this could have occurred and although this was not statistically significant, this could have significant clinical implications. For example, procedures may need to be canceled if an appropriate operating surgeon is not available.

Study limitations

This is a small sample size study in a single unit under one specialist surgeon and as such the results may not be generalizable to other units. In addition, data were not collected regarding patient outcomes following surgery so it is not possible to know if inaccurate BMI estimation affected patient outcomes. Further studies should aim to address this to confirm the validity of using self-reported BMI for general surgery operative planning.

## Conclusions

Overall we found that men tend to be more accurate at estimating their BMIs than women. We also found that women tend to under-estimate, rather than over-estimate, their BMIs. In addition, in those who under-estimate their BMIs, females do this to a greater extent than males. Both males and females with higher actual BMIs tend to be less accurate at estimating their BMIs than those with lower actual BMIs. In only two cases this could have led to a change in the operating surgeon required on the day of the procedure although this was not significant.

Therefore, overall we would conclude that estimated BMI from telephone consultations is a suitable method for assessing patients ahead of procedures, particularly for males, but it is important to be aware of the potential for inaccuracy, particularly in female patients with a higher BMI.
